# Antiamyloid therapies for Alzheimer's disease: an experts' dialogue on facts and controversies

**DOI:** 10.1055/s-0045-1808083

**Published:** 2025-05-09

**Authors:** Paulo Caramelli

**Affiliations:** 1 Universidade Federal de Minas Gerais, Faculdade de Medicina, Unidade de Neurologia Cognitiva e do Comportamento, Belo Horizonte MG, Brazil.

Dementia is a major public health problem worldwide, being highly prevalent in Latin America. 1 Alzheimer's disease (AD) is the leading cause of dementia and represents a significant challenge for patients, caregivers, and health systems, as well as for clinicians and researchers. 2

The pathogenesis of AD is complex. The pathological hallmarks of the disease are the amyloid-β (Aβ) peptide deposits and neurofibrillary tangles composed by hyperphosphorylated tau that accumulate in the brain. Over the past two decades significant advances occurred in the diagnosis of AD, particularly with the development and validation of neuroimaging and fluid-based biomarkers which allow detection of the neuropathological features of the disease. 2 3 These methods allow for earlier and more precise diagnosis, which have been fundamental for the development and testing of new therapies in the last 20 years. 4

Among the different drugs tested for AD, therapeutic strategies targeting Aβ have gained significant attention 5 and ultimately led to the approval by some regulatory agencies of three monoclonal antibodies, namely, aducanumab, donanemab, and lecanemab. 6 Aducanumab was discontinued in 2024, but donanemab and lecanemab are currently in clinical use in some countries or under evaluation by some national drug agencies.

The clinical efficacy of donanemab and lecanemab has been object of strong debate in recent years. It is well established that both drugs effectively clear amyloid deposits in the brain and significantly reduce cognitive and functional decline in comparison to placebo. 7 8 However, the magnitude or clinical relevance of the therapeutic effects, as well as the safety profile, especially the amyloid-related imaging abnormalities (ARIA), have raised concerns about the risk-benefit relationship of these drugs. Moreover, the high costs of these immunotherapies are additional obstacles for patients, families, and health systems.

This scenario is the object of discussion in the debut of the Point of View section of *Arquivos de Neuro-Psiquiatria* . Four clinicians/investigators with great experience and expertise in AD and dementia management and research provide their views on the pros and cons of antiamyloid treatment.

Jonathan Schott and Charles Marshall, from the United Kingdom, advocate for the use of these therapies, albeit with caution. The authors make their point by discussing some proven as well as possible/potential benefits of amyloid immunotherapy, and by describing the adverse events related to treatment with donanemab and lecanemab—particularly ARIA and the phenomenon they propose to be termed “amyloid related pseudoatrophy”. They also present some controversies related to these drugs and conclude in favor of their careful use in selected AD patients, in settings that are adequately prepared for treatment delivery and clinical monitoring. 9

On the other hand, Ricardo Nitrini and Adalberto Studart-Neto, from Brazil, manifest their position against the use of these new AD treatments. They review the results of the phase 3 randomized clinical trials of donanemab and lecanemab, discuss the issue of minimum clinically important difference, which was not reached, and the absence of treatment superiority in patients with mild cognitive impairment in comparison with mild dementia, as would be expected. The authors also discuss the safety profile of the two agents and the patients' eligibility for these treatments, concluding that monoclonal antibodies should not be prescribed for these individuals. 10

These two pieces offer updated, high-standard views on this controversial topic, which might be useful for clinicians, in the case these drugs are available in their settings. Many of the aspects that are discussed are of special relevance for countries with a national public health system, which is exactly the case of Brazil and the United Kingdom. Recently, the Scientific Department of Cognitive Neurology and Aging of the Brazilian Academy of Neurology (Academia Brasileira de Neurologia, ABN, in Portuguese) published a position paper about the use of antiamyloid therapies in the country. 11 The two manuscripts of this Point of View section add valuable information to this topic. Readers of *Arquivos de Neuro-Psiquiatria* shall enjoy reading these texts, which may help them form their own opinions and guide their clinical practice.
